# Limb Salvage in Patients with Concomitant Advanced Malignancy

**DOI:** 10.3390/jcm14051642

**Published:** 2025-02-28

**Authors:** Jolyn Hui Qing Pang, Nick Zhi Peng Ng

**Affiliations:** 1 Department of General Surgery, Singapore General Hospital, Outram Road, Singapore 169608, Singapore; jolyn.pang@mohh.com.sg; 2 Department of Vascular Surgery, Singapore General Hospital, Outram Road, Singapore 169608, Singapore

**Keywords:** chronic limb threatening ischemia, advanced malignancy, limb salvage

## Abstract

**Background**: We are experiencing an increasing number of patients presenting with advanced malignancy who are also presenting with chronic limb threatening ischemia. **Method**: With advancements in medicine and surgery, we are suggesting a shift in mindset of how we treat this group of patients and proposing a more holistic approach for treatment options. **Result**: In this original article, we highlight our approach in managing such patients and suggest an algorithm. **Conclusions**: We hope that our article brings awareness and assists vascular surgeons in managing such cases, resulting in improved quality of life for these patients.

## 1. Introduction

Malignancy [1] and vascular disease [2] are two of the largest disease groups in today’s age. They share many similar risk factors such as smoking and obesity [3]. Furthermore, malignancy results in a general hypercoagulable state, resulting in low flow and increased risk for thromboembolic events. This can be further exacerbated by chemotherapy [4].

Thus, it is increasingly common to encounter patients who suffer from both [5]. Nevertheless, with advances in medicine and surgery today and the ability to tailor care, neither disease is terminal, and a reasonable quality of life can be achieved.

Treatments for malignancy, even less advanced ones, often cannot commence in the presence of septic focus such as gangrenous foot, but with proper tailored therapy, meaningful limb salvage can be achieved before an upfront limb amputation.

The aim of this paper is to present two patients who, despite suffering from advanced or near terminal GI malignancy, underwent successful limb revascularization and salvage for wet gangrene and intractable rest pain, thus providing better quality end of life. In addition, the authors would like to share factors that might incline a clinician to be more favorable toward limb salvage as opposed to upfront major amputation in these patients.

## 2. Case Presentation

### 2.1. Case A

**Figure 1:**
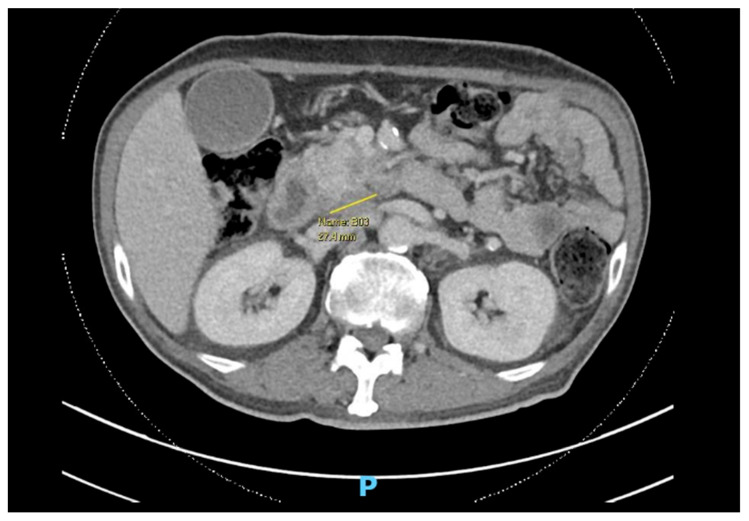
Computed Tomography (CT) scan showing axial cut of pancreatic malignancy.

Patient A is a 60-year-old male, a driver and smoker, with poorly controlled diabetes, chronic kidney disease (CKD), and ischemic heart disease (IHD), newly diagnosed with a locally advanced, borderline resectable head of pancreas malignancy with superior mesenteric vessel involvement (Figure 1).

**Figure 2:**
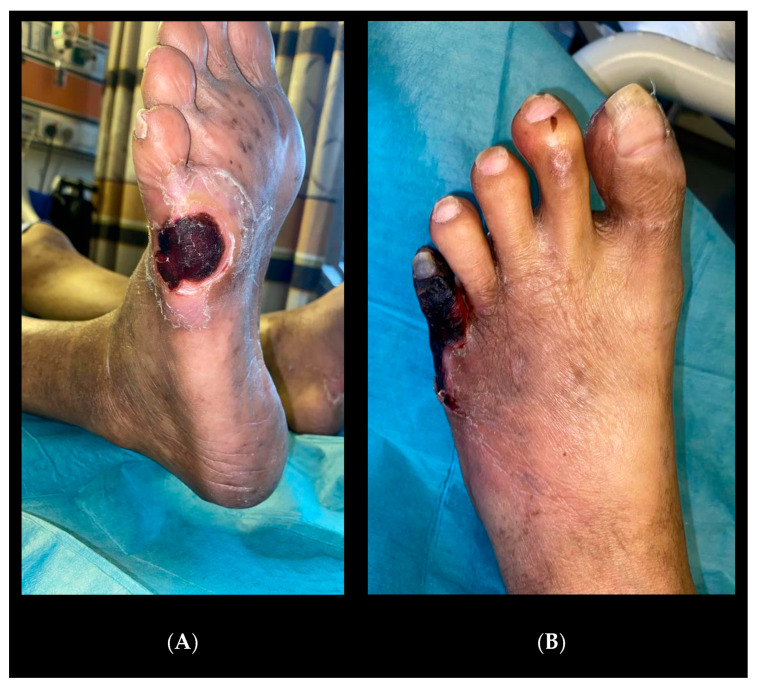
(**A**) Right foot lateral view. (**B**) Left foot dorsum view.

He was started on neoadjuvant chemotherapy with a view for the definitive Whipple procedure. He was subsequently referred to the vascular unit for bilateral fifth toe gangrene (Figure 2) that had begun as ulcers from poorly fitted shoes. He complained of increasing rest pain in the leg with purulent discharge from the left side, as well as fever, chills, and rigors.

A bilateral duplex of his lower limb arterial system was performed, and this resulted in a Wound, Ischemia, foot Infection (WIfI) score of Wound 3, Ischemia 2, Infection 2. His white cell count was 20 × 10^9^/L, and chemotherapy was held off with implications of delay in the Whipple procedure.

After a multidisciplinary discussion, the oncologist set the prognosis of his advanced untreated malignancy at less than 12 months with the cessation of treatment and likelihood of progression.

Options discussed with the patient included primary bilateral below-knee amputations (BKAs), which would allow for the best chances of healing and wound closure and for chemotherapy to be restarted sooner. Secondly was limb salvage with pedal angioplasty and bilateral fifth toe amputation, understanding the potential need for multiple debridements and failure to heal despite the above measures given his immunocompromised state, resulting in further delay to cancer treatment. Furthermore, surgery may be high risk in view of his comorbidities. Finally, the option of palliation with pain control, antibiotics, and wound dressing was discussed. The patient had difficulties accepting bilateral BKAs and was in a state of depression from the diagnosis.

**Figure 3:**
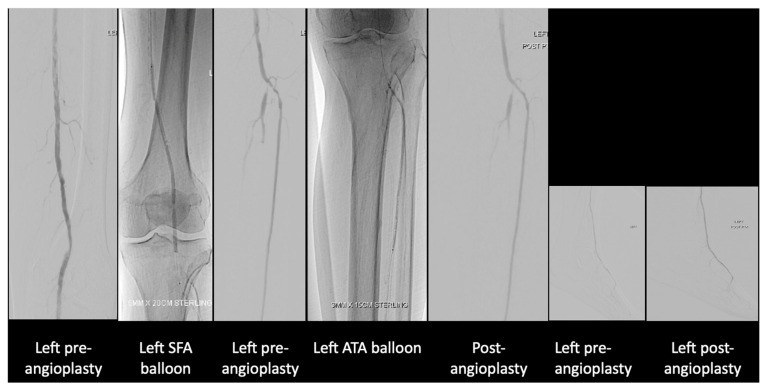
Angioplasty images of the left lower limb.

After much discussion, the patient and vascular team decided to attempt limb salvage with bilateral lower limb angioplasties and fifth toe ray amputations at the same setting. On the left lower limb, angioplasty to the anterior and posterior tibial artery was performed (Figure 3).

**Figure 4:**
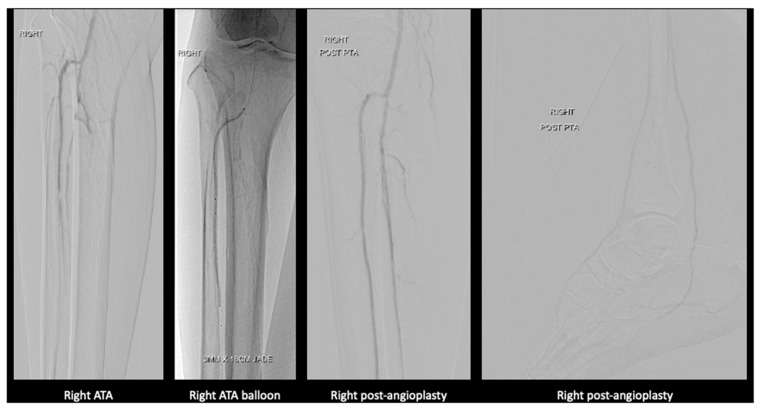
Angioplasty images of the right lower limb.

On the right lower limb, angioplasty to anterior tibial artery, posterior tibial artery, dorsalis pedis, and common plantar artery was performed (Figure 4).

**Figure 5:**
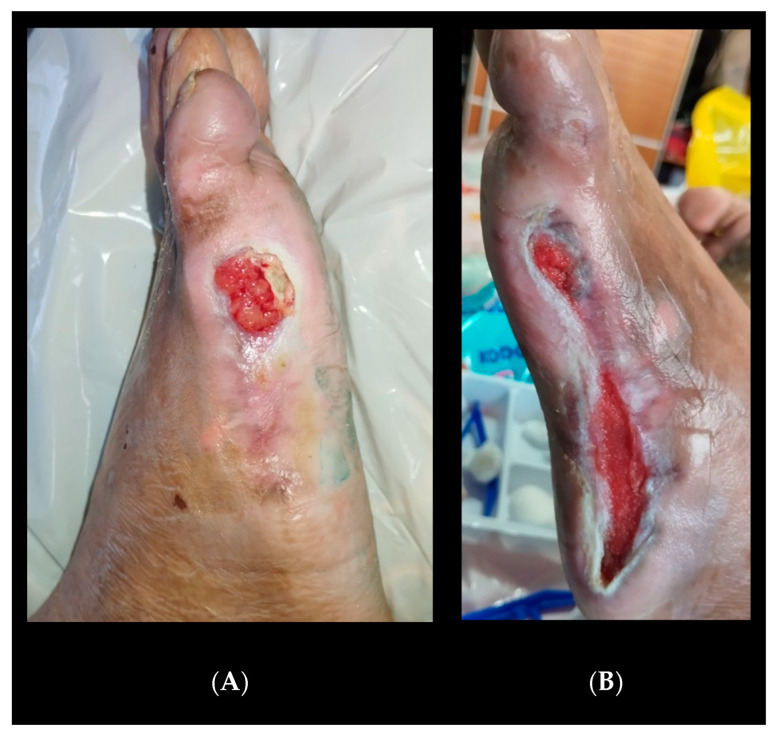
(**A**) Right foot wound. (**B**) Left foot wound.

The patient’s wound (Figure 5) recovered with a combination of culture-directed antibiotics, vacuum dressing, and motivation from a supportive family.

When the wounds showed that they had begun to granulate well and antibiotics were stopped with a normalized inflammatory marker, the oncologists resumed his chemotherapy, and he eventually underwent the Whipple procedure 3 months after the bilateral lower limb revascularization. The patient has remained well at last review, which was a year from his diagnosis.

### 2.2. Case B

Patient B is a 73-year-old male smoker of 40 pack years, with IHD and newly diagnosed gastric cancer with liver metastases and peritoneal thickening with ascites seen on imaging. He presented with transient gastric outlet obstruction that resolved with laparoscopic palliative gastrojejunal bypass and was planned for palliative chemotherapy.

**Figure 6:**
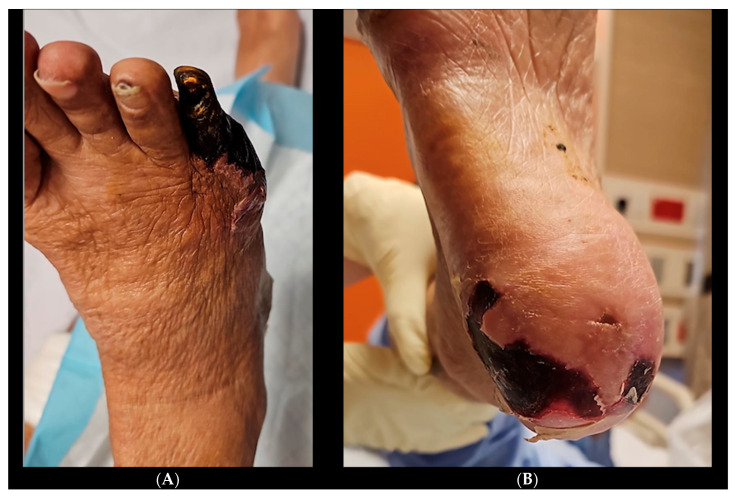
(**A**) Right foot dorsum view. (**B**) Right foot plantar view.

He had incidental right common iliac artery occlusion seen on staging scans for which he has complained, specifically of buttock and thigh claudication with occasional rest pain for more than 6 months. He now presents with right fifth toe and heel dry gangrene (Figure 6) associated with increased rest pain and inability to sleep at night despite multiple analgesics and opioids. His white cell count was 13 × 10^9^/L C-Reactive protein 38.8 mg/L.

**Figure 7:**
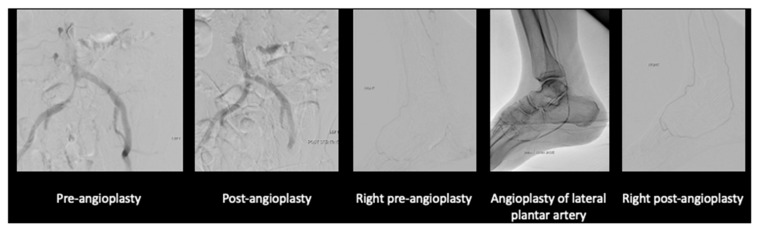
Right iliac artery and lower limb angioplasty.

Discussion was held with his family. While they noted that his life expectancy due to his malignancy was limited, their utmost concern was him having to deal with both cancer and leg pain along with the dry gangrene. His pain could not be fully palliated with the rest pain and wound in his leg. At the same time, an upfront above-knee amputation wound may not heal given the level of arterial disease. After thorough discussion, the patient and family opted for angioplasty with stenting of his right iliac artery and angioplasty of his posterior tibial artery (Figure 7).

Fortunately, the angioplasty was successful, and his fifth toe gangrene was amputated with good granulation seen.

He agreed to quit smoking and was kept on low-dose aspirin without any bleeding complications from the gastric malignancy. Unfortunately, a year later, he passed away from the progression of his cancer; however, the patient was relieved of his lower limb rest pain and could walk without claudication after the angioplasty.

## 3. Discussion

In peripheral arterial disease, there is a range of presentation, including asymptomatic disease, disabling claudication, acute limb ischemia, and chronic limb threatening ischemia (CLTI). While there is evidence to show that revascularization for acute limb ischemia in patients with cancer has acceptable short- and medium-term outcomes [6,7], there is a lack of consensus for the treatment of CLTI.

CLTI has poor long-term prognosis [8] with mortality of 17.5% at 1 year, and for those with malignancy, the prognosis is likely worse. In a cohort study by El Sakka et al. [9], CLTI patients with malignancy had a higher 6-month mortality rate of 50% compared to 20.6% in patients without malignancy. From the resource allocation point of view, patients with advanced malignancy and CLTI may be offered upfront major limb amputation in the form of below- or above-knee amputation over revascularization and limb salvage due to limited lifespan. Some may also argue that it is more humane to take a palliative route and allow patients to demise from sepsis as opposed to subjecting patients to invasive procedures, chronic wound pain, and potential future complications of underlying cancer and treatment costs. However, with advancements in medical science, vascular surgeons have an armamentarium for the treatment of CLTI, especially in the endovascular field, with overall improvement of limb salvage [10], thus preserving function in this group of patients. An endovascular approach in CLTI offers the additional benefit of reduced morbidity while achieving comparable outcomes. Furthermore, an endovascular approach in Chronic Limb-Threatening Ischemia (CLTI) offers the additional benefit of lower perioperative risk [11], which is crucial in this group of high-risk patients.

There is an increasing shift in mentality toward viewing malignancy as a chronic disease rather than a death sentence. Survival with cancer has also improved over the years [12]. The goal is for patients to be able to at least maintain an acceptable quality of life even with a diagnosis of CLTI, and this could be achieved with limb salvage as opposed to non-surgical treatment or primary amputation, which is more morbid and may be psychologically debilitating. Then, the question arises of how do we better select treatment for patients who will benefit from revascularization.

In these two cases, we demonstrated that there is still a role for lower limb revascularization and limb salvage. We propose a holistic view and identify key factors to consider and how to tailor treatment to the individual.

Firstly, we have to consider patient factors such as fitness to undergo surgery, current functional status and quality of life, and social support.

Secondly, the treatment intent of malignancy has to be clear, be it curative or palliative. If treatment is curative in intent, would CLTI hinder the treatment? For cases where treatment is palliative in intent, we have to consider whether CLTI will become a life-limiting disease process and whether it is causing symptoms. Often, CLTI can affect quality of life more than malignancy itself.

Lastly, we have to consider the feasibility of the revascularization. We can use the WIfI score to determine if limb salvage is reasonable or whether we should offer upfront amputation. If deemed salvageable, we have to consider if the angioplasty wound is technically challenging and the patient’s fitness for surgery.

**Figure 8:**
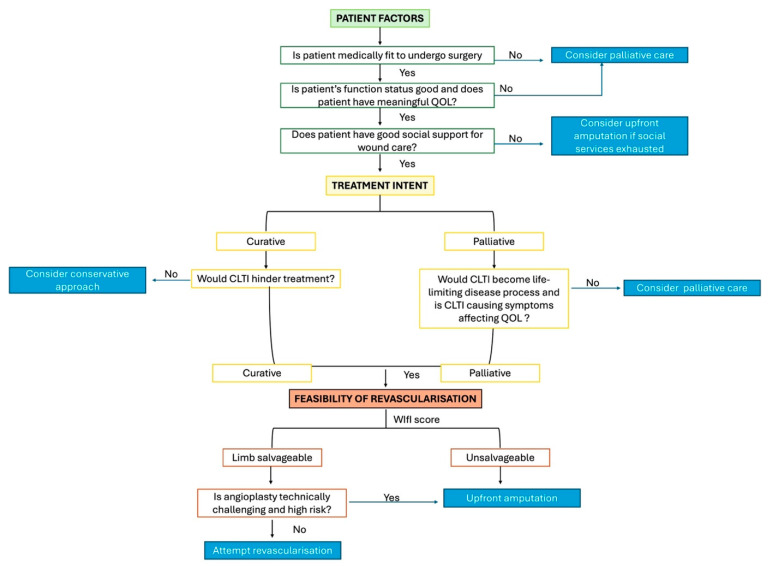
Flowsheet depicting factors to consider.

The above factors are summarized in Figure 8.

In Case A, CLTI hindered the patient’s cancer treatment. While amputation upfront may be the “best” solution to solve CLTI and allow the patient to continue chemotherapy, the patient was in a low mood as a result of the diagnosis, and this could have affected the patient’s motivation to continue his journey. However, while we would like to motivate and offer the patient a glimpse of hope with revascularization, we still had to inform the patient that limb salvage may not be successful and amputation may still be required. Delays to treatment during wound healing may also result in tumor progression and unresectability.

In Case B, the life expectancy of the patient was limited. We had to consider quality of life in his remaining days. Alleviating rest pain and regaining mobility was important to the patient. Furthermore, if revascularization was difficult and complications were expected, then we should not have proceeded as the risks may have outweighed the benefits. In this case, the patient had meaningful quality of life in his remaining days compared to if he had undergone major amputation.

## 4. Conclusions

Choosing revascularization and limb salvage over major amputation in patients with advanced malignancy can provide better quality of life despite reduced life expectancy in a selected group of patients with good patient motivation, adequate social support, effective communication regarding risks and benefits, and a supportive multidisciplinary team, including oncology, anesthesia, palliative care, and allied health teams. We hope that for those who suffer from both diseases, clinicians consider all alternatives, engage in an open conversation with the patient and family, and strive to achieve appropriate quality of life for them.

## Data Availability

The original contributions presented in this study are included in the article. Further inquiries can be directed to the corresponding author(s).
